# 4-[6,8-Dibromo-2-(2-chloro-5-nitro­phen­yl)-1,2,3,4-tetra­hydro­quinazolin-3-yl]cyclo­hexa­nol

**DOI:** 10.1107/S1600536810015023

**Published:** 2010-04-28

**Authors:** Zhi-Gang Wang, Zong-Lin Xia, Rong Wang, Ming-Li Wang

**Affiliations:** a Department of Respiratory Medicine, Third Affiliated Hospital of Soochow University, Changzhou 213003, People’s Republic of China; b Department of Pharmacy, Third Affiliated Hospital of Soochow University, Changzhou 213003, People’s Republic of China; c Modern Medical Research Center, Third Affiliated Hospital of Soochow University, Changzhou 213003, People’s Republic of China

## Abstract

The title compound, C_20_H_20_Br_2_ClN_3_O_3_, was synthesized by the condensation reaction of 2-chloro-5-nitro­benzaldehyde with 4-(2-amino-3,5-dibromo­benzyl­amino)cyclo­hexa­nol in a methanol solution. There are two independent mol­ecules in the asymmetric unit and in one mol­ecule the atoms of the cyclo­hexane ring are disordered over two sets of sites with refined occupancies of 0.657 (12) and 0.343 (12). The dihedral angle between the two benzene rings is 89.5 (2)° in one mol­ecule and 82.9 (2)° in the other. In the crystal structure, inter­molecular N—H⋯O and O—H⋯O hydrogen bonds link the mol­ecules into chains propagating along [01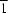].

## Related literature

For details of the pharmaceutical uses of Ambroxol, systematic name 4-(2-amino-3,5-dibromo­benzyl­amino)cyclo­hexa­nol, a compound closely related to the title compound see: Felix *et al.* (2008 ▶); Gaida *et al.* (2005 ▶); Lee *et al.* (2004 ▶). For a related structure, see: Wang *et al.* (2009 ▶). For standard bond-length data, see: Allen *et al.* (1987 ▶).
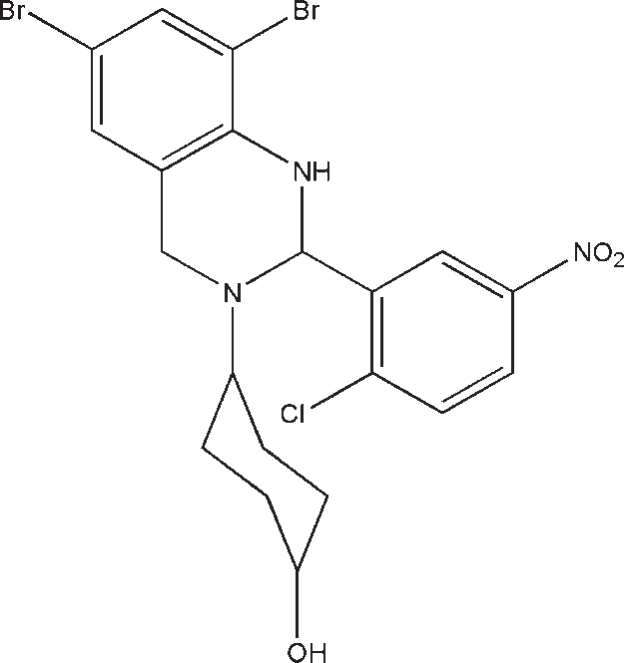


## Experimental

### 

#### Crystal data

C_20_H_20_Br_2_ClN_3_O_3_
*M* *_r_* = 545.66
Triclinic, 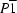

*a* = 10.2614 (13) Å
*b* = 13.1418 (17) Å
*c* = 16.931 (2) Å
α = 83.764 (2)°
β = 73.309 (2)°
γ = 84.750 (2)°
*V* = 2169.8 (5) Å^3^
*Z* = 4
Mo *K*α radiation
μ = 3.89 mm^−1^
*T* = 298 K
0.30 × 0.28 × 0.28 mm

#### Data collection

Bruker SMART CCD area-detector diffractometer
Absorption correction: multi-scan (*SADABS*; Sheldrick, 1996 ▶) *T*_min_ = 0.389, *T*_max_ = 0.409
11972 measured reflections
8470 independent reflections
4874 reflections with *I* > 2σ(*I*)
*R*_int_ = 0.022

#### Refinement

*R*[*F* ^2^ > 2σ(*F* ^2^)] = 0.055
*wR*(*F* ^2^) = 0.154
*S* = 1.02
8470 reflections
578 parameters
122 restraints
H-atom parameters constrained
Δρ_max_ = 1.56 e Å^−3^
Δρ_min_ = −1.07 e Å^−3^

### 

Data collection: *SMART* (Bruker, 2002 ▶); cell refinement: *SAINT* (Bruker, 2002 ▶); data reduction: *SAINT*; program(s) used to solve structure: *SHELXS97* (Sheldrick, 2008 ▶); program(s) used to refine structure: *SHELXL97* (Sheldrick, 2008 ▶); molecular graphics: *SHELXTL* (Sheldrick, 2008 ▶); software used to prepare material for publication: *SHELXTL*.

## Supplementary Material

Crystal structure: contains datablocks global, I. DOI: 10.1107/S1600536810015023/lh5014sup1.cif

Structure factors: contains datablocks I. DOI: 10.1107/S1600536810015023/lh5014Isup2.hkl

Additional supplementary materials:  crystallographic information; 3D view; checkCIF report

## Figures and Tables

**Table 1:** Hydrogen-bond geometry (Å, °)

*D*—H⋯*A*	*D*—H	H⋯*A*	*D*⋯*A*	*D*—H⋯*A*
N3—H3*N*⋯O4^i^	0.86	2.33	2.942 (7)	129
O3—H3⋯O6′^ii^	0.84	1.89	2.713 (12)	166
O6—H6⋯O3^iii^	0.84	2.04	2.877 (17)	179
O6′—H6′⋯O3^iii^	0.84	1.85	2.694 (10)	179

Symmetry codes: (i) 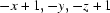; (ii) 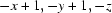; (iii) 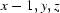.

**Fig. 1.**
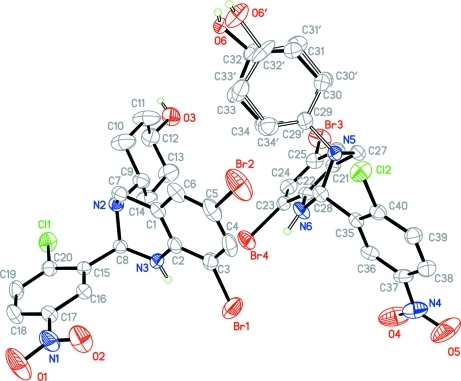
The asymmetric unit of the title compound showing 30% probability ellipsoids. H atoms bonded to C atoms are not shown.

## supplementary crystallographic information

### Comment

Ambroxol, 4-(2-amino-3,5-dibromobenzylamino)cyclohexanol, is an expectorant agent which leads to bronchial secretion due to its mucolytic properties (Felix *et al.*, 2008; Gaida *et al.*, 2005; Lee *et al.*, 2004). Recently, we reported the crystal structure of a derivative of Ambroxol (Wang *et al.*, 2009). In this paper, the crystal structure of the title compound, derived from the condensation reaction of 2-chloro-5-nitrobenzaldehyde with 4-(2-amino-3,5-dibromobenzylamino)cyclohexanol in methanol solution, is reported.

There are two independent molecules in the asymmetric unit of the title compound, Fig. 1. The dihedral angle between the two benzene rings is 89.5 (2)° in one molecule and 82.9 (2)° in the other. The cyclohexyl rings adopt chair configurations. All bond lengths are within normal ranges (Allen *et al.*, 1987).

### Experimental

2-Chloro-5-nitrobenzaldehyde (1.0 mol, 185.6 mg) and 4-(2-amino-3,5-dibromobenzylamino)cyclohexanol (1.0 mmol, 378.1 mg) were dissolved in a methanol solution (30 ml). The mixture was stirred at room temperature to give a clear colorless solution. Crystals of the title compound were formed by gradual evaporation of the solvent for a week at room temperature.

### Refinement

H atoms were included in calulated positions with, with C–H = 0.93–0.98 Å, N-H = 0.86Å and O-H = 0.84 with *U*_iso_(H) = 1.2*U*_eq_(C,N) or 1.5U_eq_(O). The C29-C34 cyclohexyl ring is disordered over two distinct sites, with refined occupancies of 0.657 (12) and 0.343 (12). Bond length restraints were applied to the disorder model using the SADI commmand in SHELXL (Sheldrick, 2008).

### Crystal data

**Table:**

C_20_H_20_Br_2_ClN_3_O_3_	*Z* = 4
*M**_r_* = 545.66	*F*(000) = 1088
Triclinic, *P*1	*D*_x_ = 1.670 Mg m^−^^3^
Hall symbol: -P 1	Mo *K*α radiation, λ = 0.71073 Å
*a* = 10.2614 (13) Å	Cell parameters from 2646 reflections
*b* = 13.1418 (17) Å	θ = 2.5–24.5°
*c* = 16.931 (2) Å	µ = 3.89 mm^−^^1^
α = 83.764 (2)°	*T* = 298 K
β = 73.309 (2)°	Block, colorless
γ = 84.750 (2)°	0.30 × 0.28 × 0.28 mm
*V* = 2169.8 (5) Å^3^	

### Data collection

**Table:**

Bruker SMART CCD area-detector diffractometer	8470 independent reflections
Radiation source: fine-focus sealed tube	4874 reflections with *I* > 2σ(*I*)
graphite	*R*_int_ = 0.022
ω scans	θ_max_ = 26.2°, θ_min_ = 1.9°
Absorption correction: multi-scan (*SADABS*; Sheldrick, 1996)	*h* = −12→7
*T*_min_ = 0.389, *T*_max_ = 0.409	*k* = −15→16
11972 measured reflections	*l* = −21→20

### Refinement

**Table:**

Refinement on *F*^2^	Primary atom site location: structure-invariant direct methods
Least-squares matrix: full	Secondary atom site location: difference Fourier map
*R*[*F*^2^ > 2σ(*F*^2^)] = 0.055	Hydrogen site location: inferred from neighbouring sites
*wR*(*F*^2^) = 0.154	H-atom parameters constrained
*S* = 1.02	*w* = 1/[σ^2^(*F*_o_^2^) + (0.0631*P*)^2^ + 2.7275*P*] where *P* = (*F*_o_^2^ + 2*F*_c_^2^)/3
8470 reflections	(Δ/σ)_max_ = 0.001
578 parameters	Δρ_max_ = 1.56 e Å^−^^3^
122 restraints	Δρ_min_ = −1.06 e Å^−^^3^

### Special details

**Table:**

Geometry. All esds (except the esd in the dihedral angle between two l.s. planes) are estimated using the full covariance matrix. The cell esds are taken into account individually in the estimation of esds in distances, angles and torsion angles; correlations between esds in cell parameters are only used when they are defined by crystal symmetry. An approximate (isotropic) treatment of cell esds is used for estimating esds involving l.s. planes.
Refinement. Refinement of F^2^ against ALL reflections. The weighted R-factor wR and goodness of fit S are based on F^2^, conventional R-factors R are based on F, with F set to zero for negative F^2^. The threshold expression of F^2^ > 2sigma(F^2^) is used only for calculating R-factors(gt) etc. and is not relevant to the choice of reflections for refinement. R-factors based on F^2^ are statistically about twice as large as those based on F, and R- factors based on ALL data will be even larger.

### Fractional atomic coordinates and isotropic or equivalent isotropic displacement parameters (Å^2^)

**Table:**

	*x*	*y*	*z*	*U*_iso_*/*U*_eq_	Occ. (<1)
Br1	0.39058 (9)	−0.05133 (6)	0.42693 (4)	0.0888 (3)	
Br2	−0.06018 (7)	0.08773 (7)	0.30819 (7)	0.1156 (4)	
Br3	0.65212 (7)	0.61194 (6)	0.35191 (6)	0.0919 (3)	
Br4	0.59092 (6)	0.18627 (5)	0.36046 (5)	0.0747 (2)	
Cl1	0.87128 (15)	−0.09542 (13)	0.01005 (10)	0.0700 (4)	
Cl2	−0.17164 (14)	0.27481 (12)	0.47738 (9)	0.0628 (4)	
O1	0.4691 (6)	−0.4725 (4)	0.1616 (4)	0.133 (3)	
O2	0.3509 (5)	−0.3554 (4)	0.2309 (4)	0.0953 (16)	
O3	0.8184 (5)	0.4244 (3)	0.0419 (3)	0.0816 (14)	
H3	0.8561	0.4573	−0.0030	0.122*	
O4	0.2375 (7)	0.1609 (5)	0.7120 (4)	0.119 (2)	
O5	0.0491 (7)	0.1570 (6)	0.8042 (4)	0.145 (3)	
N1	0.4516 (6)	−0.3856 (4)	0.1802 (4)	0.0731 (15)	
N2	0.5854 (4)	0.0384 (3)	0.0978 (3)	0.0435 (10)	
N3	0.5434 (4)	−0.0321 (3)	0.2395 (2)	0.0472 (11)	
H3N	0.5793	−0.0464	0.2797	0.057*	
N4	0.1168 (8)	0.1719 (5)	0.7331 (4)	0.0849 (18)	
N5	0.1019 (4)	0.3814 (3)	0.4122 (2)	0.0425 (10)	
N6	0.2870 (4)	0.2572 (3)	0.4237 (3)	0.0557 (12)	
H6N	0.3209	0.1949	0.4251	0.067*	
C1	0.3494 (5)	0.0295 (4)	0.1906 (3)	0.0469 (13)	
C2	0.4071 (6)	−0.0031 (4)	0.2565 (3)	0.0482 (13)	
C3	0.3189 (7)	−0.0040 (4)	0.3370 (3)	0.0579 (15)	
C4	0.1815 (7)	0.0235 (4)	0.3527 (4)	0.0704 (19)	
H4	0.1251	0.0217	0.4067	0.085*	
C5	0.1303 (6)	0.0533 (5)	0.2873 (5)	0.0723 (19)	
C6	0.2116 (6)	0.0578 (4)	0.2078 (4)	0.0598 (16)	
H6B	0.1740	0.0801	0.1646	0.072*	
C7	0.4418 (5)	0.0291 (4)	0.1042 (3)	0.0483 (13)	
H7A	0.4333	−0.0342	0.0819	0.058*	
H7B	0.4119	0.0854	0.0702	0.058*	
C8	0.6281 (5)	−0.0394 (4)	0.1557 (3)	0.0407 (12)	
H8	0.7206	−0.0254	0.1549	0.049*	
C9	0.6166 (6)	0.1452 (4)	0.1032 (4)	0.0569 (15)	
H9	0.5423	0.1758	0.1462	0.068*	
C10	0.6231 (9)	0.2061 (5)	0.0216 (5)	0.097 (3)	
H10A	0.5357	0.2064	0.0102	0.116*	
H10B	0.6912	0.1731	−0.0221	0.116*	
C11	0.6590 (10)	0.3179 (6)	0.0215 (6)	0.111 (3)	
H11A	0.6649	0.3539	−0.0324	0.133*	
H11B	0.5873	0.3528	0.0622	0.133*	
C12	0.7856 (8)	0.3205 (5)	0.0402 (4)	0.078 (2)	
H12	0.8577	0.2879	−0.0027	0.093*	
C13	0.7829 (8)	0.2642 (5)	0.1224 (5)	0.086 (2)	
H13A	0.7152	0.2978	0.1659	0.103*	
H13B	0.8711	0.2656	0.1325	0.103*	
C14	0.7484 (7)	0.1518 (5)	0.1241 (4)	0.0737 (19)	
H14A	0.8216	0.1165	0.0847	0.088*	
H14B	0.7417	0.1176	0.1787	0.088*	
C15	0.6360 (5)	−0.1458 (4)	0.1260 (3)	0.0410 (12)	
C16	0.5412 (5)	−0.2159 (4)	0.1637 (3)	0.0465 (13)	
H16	0.4664	−0.1973	0.2069	0.056*	
C17	0.5551 (6)	−0.3135 (4)	0.1386 (3)	0.0511 (13)	
C18	0.6655 (6)	−0.3457 (5)	0.0763 (4)	0.0662 (17)	
H18	0.6752	−0.4123	0.0607	0.079*	
C19	0.7607 (6)	−0.2758 (5)	0.0380 (4)	0.0614 (16)	
H19	0.8361	−0.2950	−0.0045	0.074*	
C20	0.7458 (5)	−0.1784 (4)	0.0618 (3)	0.0487 (13)	
C21	0.3193 (5)	0.4363 (4)	0.4205 (3)	0.0445 (12)	
C22	0.3731 (5)	0.3365 (4)	0.4085 (3)	0.0443 (12)	
C23	0.5137 (5)	0.3210 (4)	0.3792 (3)	0.0481 (13)	
C24	0.5984 (6)	0.4014 (5)	0.3633 (3)	0.0586 (16)	
H24	0.6925	0.3895	0.3440	0.070*	
C25	0.5415 (6)	0.4989 (5)	0.3765 (4)	0.0595 (15)	
C26	0.4024 (6)	0.5159 (4)	0.4057 (3)	0.0539 (14)	
H26	0.3647	0.5820	0.4155	0.065*	
C27	0.1651 (5)	0.4519 (4)	0.4489 (3)	0.0449 (12)	
H27A	0.1338	0.4407	0.5087	0.054*	
H27B	0.1378	0.5220	0.4331	0.054*	
C28	0.1395 (5)	0.2766 (4)	0.4373 (3)	0.0463 (13)	
H28	0.1110	0.2319	0.4034	0.056*	
O6	0.0678 (19)	0.4493 (13)	0.0811 (8)	0.057 (4)	0.343 (12)
H6	−0.0051	0.4425	0.0696	0.085*	0.343 (12)
C29	0.1191 (7)	0.4085 (5)	0.3235 (3)	0.079 (2)	0.343 (12)
H29A	0.1792	0.4634	0.3210	0.095*	0.343 (12)
C30	0.0096 (19)	0.4808 (16)	0.3083 (8)	0.052 (8)	0.343 (12)
H30A	0.0273	0.5481	0.3197	0.062*	0.343 (12)
H30B	−0.0748	0.4616	0.3485	0.062*	0.343 (12)
C31	−0.0137 (17)	0.4906 (19)	0.2262 (8)	0.067 (6)	0.343 (12)
H31A	−0.0412	0.5617	0.2137	0.080*	0.343 (12)
H31B	−0.0896	0.4502	0.2290	0.080*	0.343 (12)
C32	0.1012 (18)	0.4595 (13)	0.1567 (7)	0.065 (6)	0.343 (12)
H32A	0.1618	0.5162	0.1437	0.078*	0.343 (12)
C33	0.1854 (18)	0.3684 (13)	0.1758 (7)	0.053 (6)	0.343 (12)
H33A	0.1398	0.3080	0.1731	0.063*	0.343 (12)
H33B	0.2713	0.3671	0.1327	0.063*	0.343 (12)
C34	0.2156 (15)	0.3599 (13)	0.2562 (6)	0.057 (5)	0.343 (12)
H34A	0.2257	0.2876	0.2737	0.068*	0.343 (12)
H34B	0.3032	0.3881	0.2476	0.068*	0.343 (12)
O6'	0.0198 (12)	0.4810 (10)	0.0975 (6)	0.095 (4)	0.657 (12)
H6'	−0.0436	0.4642	0.0802	0.142*	0.657 (12)
C29'	0.1191 (7)	0.4085 (5)	0.3235 (3)	0.079 (2)	0.657 (12)
H29B	0.2138	0.4277	0.3026	0.095*	0.657 (12)
C30'	0.0385 (12)	0.5058 (7)	0.3093 (5)	0.049 (3)	0.657 (12)
H30C	0.0738	0.5610	0.3292	0.059*	0.657 (12)
H30D	−0.0552	0.4992	0.3425	0.059*	0.657 (12)
C31'	0.0389 (12)	0.5356 (7)	0.2214 (5)	0.067 (3)	0.657 (12)
H31C	−0.0314	0.5897	0.2198	0.081*	0.657 (12)
H31D	0.1262	0.5619	0.1908	0.081*	0.657 (12)
C32'	0.0143 (13)	0.4461 (8)	0.1814 (6)	0.081 (4)	0.657 (12)
H32B	−0.0764	0.4224	0.2101	0.098*	0.657 (12)
C33'	0.1193 (15)	0.3608 (7)	0.1855 (5)	0.077 (4)	0.657 (12)
H33C	0.2091	0.3839	0.1565	0.092*	0.657 (12)
H33D	0.1037	0.3037	0.1583	0.092*	0.657 (12)
C34'	0.1142 (13)	0.3259 (6)	0.2735 (5)	0.072 (3)	0.657 (12)
H34C	0.0309	0.2912	0.2989	0.087*	0.657 (12)
H34D	0.1902	0.2764	0.2739	0.087*	0.657 (12)
C35	0.0602 (5)	0.2507 (4)	0.5269 (3)	0.0420 (12)	
C36	0.1226 (6)	0.2241 (4)	0.5892 (4)	0.0519 (14)	
H36	0.2172	0.2205	0.5769	0.062*	
C37	0.0451 (7)	0.2028 (4)	0.6695 (4)	0.0570 (15)	
C38	−0.0942 (7)	0.2086 (4)	0.6919 (4)	0.0617 (16)	
H38	−0.1444	0.1964	0.7469	0.074*	
C39	−0.1569 (6)	0.2328 (4)	0.6310 (3)	0.0516 (14)	
H39	−0.2515	0.2360	0.6442	0.062*	
C40	−0.0820 (5)	0.2523 (4)	0.5505 (3)	0.0435 (12)	

### Atomic displacement parameters (Å^2^)

**Table:**

	*U*^11^	*U*^22^	*U*^33^	*U*^12^	*U*^13^	*U*^23^
Br1	0.1211 (7)	0.0938 (6)	0.0491 (4)	−0.0380 (5)	−0.0139 (4)	0.0028 (3)
Br2	0.0488 (4)	0.0931 (6)	0.1853 (10)	0.0068 (4)	0.0052 (5)	−0.0404 (6)
Br3	0.0649 (4)	0.0829 (5)	0.1281 (7)	−0.0302 (4)	−0.0261 (4)	0.0094 (5)
Br4	0.0500 (4)	0.0687 (4)	0.0959 (5)	0.0196 (3)	−0.0086 (3)	−0.0173 (4)
Cl1	0.0529 (9)	0.0799 (11)	0.0661 (10)	−0.0119 (8)	0.0041 (8)	−0.0084 (8)
Cl2	0.0496 (8)	0.0840 (11)	0.0619 (9)	−0.0129 (7)	−0.0264 (7)	−0.0010 (8)
O1	0.120 (5)	0.060 (3)	0.193 (7)	−0.028 (3)	0.019 (4)	−0.048 (4)
O2	0.073 (3)	0.074 (3)	0.120 (4)	−0.024 (3)	0.013 (3)	−0.020 (3)
O3	0.106 (4)	0.048 (3)	0.083 (3)	−0.029 (2)	−0.010 (3)	0.002 (2)
O4	0.096 (4)	0.143 (5)	0.134 (5)	−0.020 (4)	−0.076 (4)	0.052 (4)
O5	0.147 (6)	0.219 (8)	0.072 (4)	−0.013 (5)	−0.056 (4)	0.040 (4)
N1	0.073 (4)	0.048 (3)	0.094 (4)	−0.014 (3)	−0.011 (3)	−0.012 (3)
N2	0.046 (2)	0.038 (2)	0.048 (3)	−0.0040 (19)	−0.015 (2)	−0.004 (2)
N3	0.055 (3)	0.051 (3)	0.037 (2)	0.004 (2)	−0.017 (2)	−0.006 (2)
N4	0.099 (5)	0.081 (4)	0.085 (5)	−0.012 (4)	−0.052 (4)	0.022 (3)
N5	0.040 (2)	0.045 (3)	0.042 (2)	0.0008 (19)	−0.0101 (19)	−0.0040 (19)
N6	0.042 (3)	0.038 (3)	0.079 (3)	0.006 (2)	−0.005 (2)	−0.010 (2)
C1	0.047 (3)	0.036 (3)	0.057 (3)	−0.001 (2)	−0.014 (3)	−0.009 (3)
C2	0.059 (3)	0.031 (3)	0.053 (3)	−0.008 (2)	−0.008 (3)	−0.010 (2)
C3	0.076 (4)	0.040 (3)	0.050 (3)	−0.014 (3)	−0.002 (3)	−0.006 (3)
C4	0.068 (4)	0.050 (4)	0.072 (5)	−0.015 (3)	0.023 (4)	−0.021 (3)
C5	0.049 (4)	0.055 (4)	0.101 (6)	0.001 (3)	0.003 (4)	−0.022 (4)
C6	0.052 (3)	0.044 (3)	0.084 (5)	0.002 (3)	−0.015 (3)	−0.016 (3)
C7	0.049 (3)	0.043 (3)	0.056 (3)	0.002 (2)	−0.021 (3)	−0.004 (3)
C8	0.040 (3)	0.040 (3)	0.044 (3)	−0.001 (2)	−0.016 (2)	−0.003 (2)
C9	0.063 (4)	0.048 (3)	0.063 (4)	−0.005 (3)	−0.023 (3)	−0.001 (3)
C10	0.142 (7)	0.068 (5)	0.106 (6)	−0.036 (5)	−0.080 (6)	0.023 (4)
C11	0.148 (8)	0.072 (5)	0.141 (8)	−0.035 (5)	−0.094 (7)	0.039 (5)
C12	0.099 (5)	0.060 (4)	0.070 (4)	−0.031 (4)	−0.012 (4)	0.005 (3)
C13	0.110 (6)	0.073 (5)	0.088 (5)	−0.035 (4)	−0.045 (5)	0.004 (4)
C14	0.079 (5)	0.067 (4)	0.082 (5)	−0.022 (4)	−0.037 (4)	0.014 (4)
C15	0.043 (3)	0.041 (3)	0.040 (3)	0.001 (2)	−0.014 (2)	−0.001 (2)
C16	0.047 (3)	0.044 (3)	0.048 (3)	0.001 (2)	−0.013 (3)	−0.005 (2)
C17	0.052 (3)	0.041 (3)	0.059 (4)	−0.002 (3)	−0.014 (3)	−0.006 (3)
C18	0.070 (4)	0.047 (4)	0.078 (4)	0.007 (3)	−0.012 (4)	−0.021 (3)
C19	0.056 (4)	0.065 (4)	0.052 (4)	0.003 (3)	0.004 (3)	−0.018 (3)
C20	0.043 (3)	0.051 (3)	0.048 (3)	0.000 (2)	−0.008 (3)	−0.004 (3)
C21	0.044 (3)	0.042 (3)	0.046 (3)	0.002 (2)	−0.014 (2)	0.000 (2)
C22	0.045 (3)	0.042 (3)	0.044 (3)	0.003 (2)	−0.013 (2)	0.001 (2)
C23	0.041 (3)	0.056 (3)	0.044 (3)	0.004 (3)	−0.010 (2)	−0.003 (3)
C24	0.036 (3)	0.081 (5)	0.058 (4)	−0.001 (3)	−0.016 (3)	0.003 (3)
C25	0.049 (3)	0.062 (4)	0.066 (4)	−0.012 (3)	−0.016 (3)	0.004 (3)
C26	0.053 (3)	0.050 (3)	0.058 (4)	−0.005 (3)	−0.014 (3)	−0.003 (3)
C27	0.041 (3)	0.036 (3)	0.052 (3)	−0.001 (2)	−0.006 (3)	−0.004 (2)
C28	0.042 (3)	0.043 (3)	0.054 (3)	−0.002 (2)	−0.013 (3)	−0.007 (3)
O6	0.080 (8)	0.053 (7)	0.038 (7)	0.013 (6)	−0.021 (6)	−0.010 (5)
C29	0.095 (5)	0.086 (5)	0.056 (4)	0.039 (4)	−0.028 (4)	−0.021 (4)
C30	0.055 (10)	0.053 (10)	0.045 (10)	0.000 (8)	−0.011 (7)	−0.007 (7)
C31	0.072 (10)	0.074 (10)	0.056 (9)	0.013 (8)	−0.027 (8)	−0.002 (8)
C32	0.099 (18)	0.061 (13)	0.038 (11)	0.000 (12)	−0.025 (12)	−0.005 (9)
C33	0.040 (11)	0.080 (14)	0.038 (10)	−0.002 (9)	−0.010 (8)	−0.009 (9)
C34	0.045 (10)	0.070 (12)	0.053 (11)	0.010 (9)	−0.013 (9)	−0.009 (9)
O6'	0.114 (7)	0.125 (8)	0.053 (5)	−0.037 (6)	−0.030 (5)	0.003 (5)
C29'	0.095 (5)	0.086 (5)	0.056 (4)	0.039 (4)	−0.028 (4)	−0.021 (4)
C30'	0.060 (6)	0.043 (6)	0.050 (6)	−0.006 (5)	−0.021 (5)	−0.009 (4)
C31'	0.066 (6)	0.076 (6)	0.063 (6)	−0.006 (5)	−0.027 (5)	0.004 (5)
C32'	0.079 (9)	0.139 (12)	0.029 (6)	−0.050 (9)	−0.015 (6)	0.020 (6)
C33'	0.103 (9)	0.077 (7)	0.047 (6)	−0.022 (6)	−0.009 (6)	−0.011 (5)
C34'	0.092 (7)	0.061 (6)	0.067 (6)	−0.012 (5)	−0.027 (5)	0.003 (5)
C35	0.042 (3)	0.036 (3)	0.050 (3)	0.002 (2)	−0.017 (3)	−0.009 (2)
C36	0.046 (3)	0.041 (3)	0.069 (4)	0.003 (2)	−0.022 (3)	0.001 (3)
C37	0.075 (4)	0.046 (3)	0.058 (4)	−0.002 (3)	−0.036 (3)	0.007 (3)
C38	0.075 (4)	0.050 (4)	0.055 (4)	−0.005 (3)	−0.013 (3)	0.004 (3)
C39	0.049 (3)	0.054 (3)	0.050 (3)	−0.002 (3)	−0.014 (3)	0.000 (3)
C40	0.044 (3)	0.040 (3)	0.051 (3)	−0.001 (2)	−0.020 (3)	−0.005 (2)

### Geometric parameters (Å, °)

**Table:**

Br1—C3	1.897 (6)	C18—H18	0.9300
Br2—C5	1.904 (6)	C19—C20	1.365 (8)
Br3—C25	1.890 (6)	C19—H19	0.9300
Br4—C23	1.893 (5)	C21—C26	1.367 (7)
Cl1—C20	1.737 (5)	C21—C22	1.387 (7)
Cl2—C40	1.729 (5)	C21—C27	1.517 (7)
O1—N1	1.201 (6)	C22—C23	1.388 (7)
O2—N1	1.204 (7)	C23—C24	1.384 (8)
O3—C12	1.441 (7)	C24—C25	1.373 (8)
O3—H3	0.8401	C24—H24	0.9300
O4—N4	1.186 (8)	C25—C26	1.375 (8)
O5—N4	1.210 (8)	C26—H26	0.9300
N1—C17	1.460 (7)	C27—H27A	0.9700
N2—C7	1.461 (6)	C27—H27B	0.9700
N2—C8	1.464 (6)	C28—C35	1.519 (7)
N2—C9	1.487 (7)	C28—H28	0.9800
N3—C2	1.371 (7)	O6—C32	1.439 (10)
N3—C8	1.444 (6)	O6—H6	0.8401
N3—H3N	0.8600	O6—H6'	1.1466
N4—C37	1.472 (8)	C29—C34	1.442 (8)
N5—C28	1.448 (6)	C29—C30	1.466 (8)
N5—C27	1.460 (6)	C29—H29A	0.9800
N5—C29	1.470 (7)	C30—C31	1.468 (9)
N6—C22	1.383 (6)	C30—H30A	0.9700
N6—C28	1.467 (6)	C30—H30B	0.9700
N6—H6N	0.8600	C31—C32	1.469 (9)
C1—C6	1.383 (7)	C31—H31A	0.9700
C1—C2	1.416 (7)	C31—H31B	0.9700
C1—C7	1.499 (7)	C32—C33	1.474 (9)
C2—C3	1.403 (8)	C32—H32A	0.9800
C3—C4	1.379 (9)	C33—C34	1.470 (9)
C4—C5	1.364 (9)	C33—H33A	0.9700
C4—H4	0.9300	C33—H33B	0.9700
C5—C6	1.363 (9)	C34—H34A	0.9700
C6—H6B	0.9300	C34—H34B	0.9700
C7—H7A	0.9700	O6'—C32'	1.432 (12)
C7—H7B	0.9700	O6'—H6	0.8290
C8—C15	1.525 (7)	O6'—H6'	0.8401
C8—H8	0.9800	C30'—C31'	1.496 (7)
C9—C14	1.505 (8)	C30'—H30C	0.9700
C9—C10	1.507 (8)	C30'—H30D	0.9700
C9—H9	0.9800	C31'—C32'	1.494 (7)
C10—C11	1.548 (9)	C31'—H31C	0.9700
C10—H10A	0.9700	C31'—H31D	0.9700
C10—H10B	0.9700	C32'—C33'	1.492 (8)
C11—C12	1.427 (10)	C32'—H32B	0.9800
C11—H11A	0.9700	C33'—C34'	1.497 (7)
C11—H11B	0.9700	C33'—H33C	0.9700
C12—C13	1.497 (9)	C33'—H33D	0.9700
C12—H12	0.9800	C34'—H34C	0.9700
C13—C14	1.545 (9)	C34'—H34D	0.9700
C13—H13A	0.9700	C35—C36	1.382 (7)
C13—H13B	0.9700	C35—C40	1.397 (7)
C14—H14A	0.9700	C36—C37	1.377 (8)
C14—H14B	0.9700	C36—H36	0.9300
C15—C16	1.372 (7)	C37—C38	1.367 (8)
C15—C20	1.394 (7)	C38—C39	1.360 (8)
C16—C17	1.376 (7)	C38—H38	0.9300
C16—H16	0.9300	C39—C40	1.369 (7)
C17—C18	1.378 (8)	C39—H39	0.9300
C18—C19	1.375 (8)		
			
C12—O3—H3	118.6	C24—C23—C22	121.7 (5)
O1—N1—O2	122.5 (6)	C24—C23—Br4	119.4 (4)
O1—N1—C17	118.7 (6)	C22—C23—Br4	118.9 (4)
O2—N1—C17	118.8 (5)	C25—C24—C23	119.0 (5)
C7—N2—C8	109.3 (4)	C25—C24—H24	120.5
C7—N2—C9	112.8 (4)	C23—C24—H24	120.5
C8—N2—C9	115.5 (4)	C24—C25—C26	120.2 (5)
C2—N3—C8	121.6 (4)	C24—C25—Br3	120.6 (4)
C2—N3—H3N	119.2	C26—C25—Br3	119.1 (5)
C8—N3—H3N	119.2	C21—C26—C25	120.5 (5)
O4—N4—O5	123.2 (7)	C21—C26—H26	119.8
O4—N4—C37	118.6 (7)	C25—C26—H26	119.8
O5—N4—C37	118.1 (7)	N5—C27—C21	110.9 (4)
C28—N5—C27	109.7 (4)	N5—C27—H27A	109.5
C28—N5—C29	117.3 (4)	C21—C27—H27A	109.5
C27—N5—C29	112.7 (4)	N5—C27—H27B	109.5
C22—N6—C28	121.7 (4)	C21—C27—H27B	109.5
C22—N6—H6N	119.2	H27A—C27—H27B	108.1
C28—N6—H6N	119.2	N5—C28—N6	112.7 (4)
C6—C1—C2	119.6 (5)	N5—C28—C35	109.0 (4)
C6—C1—C7	122.8 (5)	N6—C28—C35	112.2 (4)
C2—C1—C7	117.6 (5)	N5—C28—H28	107.6
N3—C2—C3	123.3 (5)	N6—C28—H28	107.6
N3—C2—C1	119.7 (5)	C35—C28—H28	107.6
C3—C2—C1	117.0 (5)	C32—O6—H6	134.0
C4—C3—C2	122.4 (6)	C32—O6—H6'	118.5
C4—C3—Br1	119.0 (5)	H6—O6—H6'	20.8
C2—C3—Br1	118.5 (5)	C34—C29—C30	120.7 (6)
C5—C4—C3	118.5 (6)	C34—C29—N5	126.4 (6)
C5—C4—H4	120.8	C30—C29—N5	112.1 (6)
C3—C4—H4	120.8	C34—C29—H29A	93.0
C6—C5—C4	121.6 (6)	C30—C29—H29A	93.0
C6—C5—Br2	119.5 (6)	N5—C29—H29A	93.0
C4—C5—Br2	118.8 (5)	C29—C30—C31	118.7 (7)
C5—C6—C1	120.8 (6)	C29—C30—H30A	107.6
C5—C6—H6B	119.6	C31—C30—H30A	107.6
C1—C6—H6B	119.6	C29—C30—H30B	107.6
N2—C7—C1	114.2 (4)	C31—C30—H30B	107.6
N2—C7—H7A	108.7	H30A—C30—H30B	107.1
C1—C7—H7A	108.7	C30—C31—C32	117.0 (8)
N2—C7—H7B	108.7	C30—C31—H31A	108.0
C1—C7—H7B	108.7	C32—C31—H31A	108.1
H7A—C7—H7B	107.6	C30—C31—H31B	108.0
N3—C8—N2	111.8 (4)	C32—C31—H31B	108.0
N3—C8—C15	113.2 (4)	H31A—C31—H31B	107.3
N2—C8—C15	110.3 (4)	O6—C32—C31	115.5 (14)
N3—C8—H8	107.1	O6—C32—C33	109.5 (12)
N2—C8—H8	107.1	C31—C32—C33	115.2 (9)
C15—C8—H8	107.1	O6—C32—H32A	105.1
N2—C9—C14	113.4 (5)	C31—C32—H32A	105.1
N2—C9—C10	108.7 (5)	C33—C32—H32A	105.1
C14—C9—C10	109.1 (5)	C34—C33—C32	117.0 (8)
N2—C9—H9	108.5	C34—C33—H33A	108.1
C14—C9—H9	108.5	C32—C33—H33A	108.1
C10—C9—H9	108.5	C34—C33—H33B	108.1
C9—C10—C11	111.9 (6)	C32—C33—H33B	108.1
C9—C10—H10A	109.2	H33A—C33—H33B	107.3
C11—C10—H10A	109.2	C29—C34—C33	118.0 (7)
C9—C10—H10B	109.2	C29—C34—H34A	107.8
C11—C10—H10B	109.2	C33—C34—H34A	107.8
H10A—C10—H10B	107.9	C29—C34—H34B	107.8
C12—C11—C10	110.9 (6)	C33—C34—H34B	107.8
C12—C11—H11A	109.5	H34A—C34—H34B	107.1
C10—C11—H11A	109.5	C32'—O6'—H6	118.2
C12—C11—H11B	109.5	C32'—O6'—H6'	116.1
C10—C11—H11B	109.5	H6—O6'—H6'	32.6
H11A—C11—H11B	108.1	C31'—C30'—H30C	108.4
C11—C12—O3	111.2 (6)	C31'—C30'—H30D	108.4
C11—C12—C13	111.6 (6)	H30C—C30'—H30D	107.5
O3—C12—C13	108.1 (5)	C32'—C31'—C30'	111.1 (7)
C11—C12—H12	108.6	C32'—C31'—H31C	109.4
O3—C12—H12	108.6	C30'—C31'—H31C	109.4
C13—C12—H12	108.6	C32'—C31'—H31D	109.4
C12—C13—C14	110.3 (5)	C30'—C31'—H31D	109.4
C12—C13—H13A	109.6	H31C—C31'—H31D	108.0
C14—C13—H13A	109.6	O6'—C32'—C33'	111.3 (9)
C12—C13—H13B	109.6	O6'—C32'—C31'	107.2 (9)
C14—C13—H13B	109.6	C33'—C32'—C31'	110.1 (7)
H13A—C13—H13B	108.1	O6'—C32'—H32B	109.4
C9—C14—C13	111.8 (5)	C33'—C32'—H32B	109.4
C9—C14—H14A	109.3	C31'—C32'—H32B	109.4
C13—C14—H14A	109.3	C32'—C33'—C34'	110.9 (7)
C9—C14—H14B	109.3	C32'—C33'—H33C	109.5
C13—C14—H14B	109.3	C34'—C33'—H33C	109.5
H14A—C14—H14B	107.9	C32'—C33'—H33D	109.5
C16—C15—C20	116.8 (5)	C34'—C33'—H33D	109.5
C16—C15—C8	122.2 (5)	H33C—C33'—H33D	108.1
C20—C15—C8	120.8 (5)	C33'—C34'—H34C	108.7
C15—C16—C17	120.9 (5)	C33'—C34'—H34D	108.7
C15—C16—H16	119.5	H34C—C34'—H34D	107.6
C17—C16—H16	119.5	C36—C35—C40	116.3 (5)
C16—C17—C18	121.8 (5)	C36—C35—C28	122.9 (5)
C16—C17—N1	119.2 (5)	C40—C35—C28	120.9 (4)
C18—C17—N1	119.0 (5)	C37—C36—C35	120.2 (5)
C19—C18—C17	117.6 (5)	C37—C36—H36	119.9
C19—C18—H18	121.2	C35—C36—H36	119.9
C17—C18—H18	121.2	C38—C37—C36	122.7 (5)
C20—C19—C18	120.7 (5)	C38—C37—N4	119.3 (6)
C20—C19—H19	119.7	C36—C37—N4	118.0 (6)
C18—C19—H19	119.7	C39—C38—C37	117.7 (6)
C19—C20—C15	122.1 (5)	C39—C38—H38	121.1
C19—C20—Cl1	117.5 (4)	C37—C38—H38	121.1
C15—C20—Cl1	120.4 (4)	C38—C39—C40	120.7 (5)
C26—C21—C22	121.0 (5)	C38—C39—H39	119.7
C26—C21—C27	122.4 (5)	C40—C39—H39	119.7
C22—C21—C27	116.7 (5)	C39—C40—C35	122.4 (5)
N6—C22—C21	120.0 (5)	C39—C40—Cl2	116.8 (4)
N6—C22—C23	122.3 (5)	C35—C40—Cl2	120.8 (4)
C21—C22—C23	117.7 (5)		

### Hydrogen-bond geometry (Å, °)

**Table:**

*D*—H···*A*	*D*—H	H···*A*	*D*···*A*	*D*—H···*A*
N3—H3N···O4^i^	0.86	2.33	2.942 (7)	129
O3—H3···O6'^ii^	0.84	1.89	2.713 (12)	166
O6—H6···O3^iii^	0.84	2.04	2.877 (17)	179
O6'—H6'···O3^iii^	0.84	1.85	2.694 (10)	179

Symmetry codes: (i) −*x*+1, −*y*, −*z*+1; (ii) −*x*+1, −*y*+1, −*z*; (iii) *x*−1, *y*, *z*.

## References

[bb1] Allen, F. H., Kennard, O., Watson, D. G., Brammer, L., Orpen, A. G. & Taylor, R. (1987). *J. Chem. Soc. Perkin Trans. 2*, pp. S1–19.

[bb2] Bruker (2002). *SAINT* and *SMART* Bruker AXS Inc., Madison, Wisconsin, USA.

[bb3] Felix, F. S., Brett, C. M. A. & Angnes, L. (2008). *Talanta*, **76**, 128–133. 10.1016/j.talanta.2008.02.005 18585253

[bb4] Gaida, W., Klinder, K., Arndt, K. & Weiser, T. (2005). *Neuropharmacology*, **49**, 1220–1227. 10.1016/j.neuropharm.2005.08.004 16182323

[bb5] Lee, H. J., Joung, S. K., Kim, Y. G., Yoo, J.-Y. & Han, S. B. (2004). *Pharm. Res.* **49**, 93–98. 10.1016/j.phrs.2003.07.011 14597158

[bb6] Sheldrick, G. M. (1996). *SADABS* University of Göttingen, Germany.

[bb7] Sheldrick, G. M. (2008). *Acta Cryst.* A**64**, 112–122. 10.1107/S0108767307043930 18156677

[bb8] Wang, Z.-G., Wang, R., Zhang, Y., Zhi, F. & Yang, Y.-L. (2009). *Acta Cryst.* E**65**, o550. 10.1107/S1600536809005182 PMC2968568 21582209

